# Lectures on the Operations of Surgery, and on Diseases and Accidents Requiring Operations

**Published:** 1846-03

**Authors:** 


ART. XI.—*Lectures on the Operations of Surgery, and on Diseases and Accidents requiring Operations.* By ROBERT LISTON, Esq., F. R. S., Senior Surgeon to the University College Hospital, and Prof. of Clinical Surgery in the College. *With numerous Additions.* By THOMAS D. MUTTER, M. D., Professor of Surgery in Jefferson Medical College, Philadelphia; Fellow of the Royal Medical and Chirurgical Society of London; Foreign Honorary and Corresponding Member of the Provincial Medical Association of Great Britain; Corresponding Member of the National Institute at Washington; Fellow of the College of Physicians, Philadelphia; Member of the Academy of Natural Sciences, Philadelphia; Corresponding Member of the New York Medical Society; Honorary Member of the Medical Societies of Philadelphia, Virginia, &c. &c. &c. Philadelphia: Lea & Blanchard. 1846. (8vo. pp. 565.)

These Lectures were delivered in 1844, at the University College, London, and published in the London Lancet. Dr. Mütter has rendered a service to his professional brethren in this country by their reproduction in the present form.

This volume embraces all the lectures of Mr. Liston published up to this time, but does not conclude the course. It will be followed by a second, should the publication of the lectures be continued. The additions of Dr. Mütter make nearly one-half of the volume. We have barely room to announce its publication here; but shall seize an early opportunity to notice it at greater length.

